# The anti-tumor immune response generated by radiation therapy may be limited by tumor cell adaptive resistance and can be circumvented by PD-L1 blockade

**DOI:** 10.1186/2051-1426-2-S3-O9

**Published:** 2014-11-06

**Authors:** Simon Dovedi, Amy Adlard, Grazyna Lipowska-Bhalla, Conor McKenna, Sherrie Jones, Eleanor Cheadle, Ian Stratford, Edmund Poon, Michelle Morrow, Ross Stewart, Hazel Jones, Robert W Wilkinson, Jamie Honeychurch, Tim Illidge

**Affiliations:** 1 University of Manchester, Manchester, United Kingdom; 2 Institute of Cancer Sciences, University of Manchester, Manchester, United Kingdom; 3 MedImmune, Cambridge, United Kingdom; 4 Cancer Research UK, London, United Kingdom

## 

Radiation therapy (RT) plays a definitive part of anti-cancer therapy for the majority of common cancers but for many patients, metastatic disease and local recurrence are common and the outlook remains poor. New more effective RT combination approaches are urgently required that decrease local and distant recurrence to improve outcomes.

Using a range of established syngeneic tumor models (CT26 (colorectal), 4T1 (breast) or 4434 (BRafV600E p16^-/- ^melanoma) we sought to determine the impact of fractionated RT (fRT) on the tumor microenvironment. Our data reveal that treatment with a course of fRT leads to significant upregulation of tumor cell expression of PD-L1 *in vivo*. Through cellular depletion studies we determined that this RT-mediated increase in tumor cell expression of PD-L1 was dependent on CD8^+ ^T cells. Furthermore, ShRNA-mediated silencing of tumor cell IFNγR1 expression or administration of an IFNγ depleting mAb phenocopied the depletion of CD8^+ ^T cells. Taken together, these data demonstrate that adaptive upregulation of tumor cell PD-L1 following treatment with low-dose fRT is mediated by tumor infiltrating CD8^+ ^T cell production of IFNγ. Using a dual tumor model our data reveal that this adaptive upregulation is restricted to the irradiated tumor site with no change in tumor cell PD-L1 expression detected in tumors situated outside of the ionizing radiation field, signifying that treatment with RT alone may not generate systemic tumor antigen-specific responses. Administration of either an anti-PD-1 or anti-PD-L1 mAb in combination with fRT was found to substantially improve survival when compared to either monotherapy alone. In addition, abscopal responses were observed on tumors outside of the RT treatment field. Our data reveal that up to 60% of mice undergo a complete response following combination therapy and are protected against tumor rechallenge by the generation of long-term immunological memory. Furthermore, we found that scheduling of anti-PD-L1 mAb relative to the delivery of fRT appeared important to therapeutic outcome with concomitant but not sequential administration required for improved survival.

Tumor cell PD-L1 expression following treatment with fRT appears to be a mechanism of adaptive immunological resistance which may potentially contribute to fRT treatment failure. This study demonstrates the potential for enhancing the efficacy of conventional RT through blockade of the PD-1/PD-L1 axis, but sequencing is critical, providing important new insights for clinical evaluation.

